# Don’t Go Breaking My Heart: MCMV as a Model for HCMV-Associated Cardiovascular Diseases

**DOI:** 10.3390/pathogens10050619

**Published:** 2021-05-18

**Authors:** Cassandra M. Bonavita, Rhonda D. Cardin

**Affiliations:** Department of Pathobiological Sciences, School of Veterinary Medicine, Louisiana State University, Baton Rouge, LA 70803, USA; cbonav6@lsu.edu

**Keywords:** cytomegalovirus, viral pathogenesis, co-morbidity, host-pathogen interaction, organ manifestation, cardiovascular diseases

## Abstract

Human Cytomegalovirus (HCMV) is a widespread pathogen that causes lifelong latent infection and is associated with the exacerbation of chronic inflammatory diseases in seropositive individuals. Of particular impact, HCMV infection is known to worsen many cardiovascular diseases including myocarditis, atherosclerosis, hypertension, and transplant vasculopathy. Due to its similarity to HCMV, murine CMV (MCMV) is an appropriate model to understand HCMV-induced pathogenesis in the heart and vasculature. MCMV shares similar sequence homology and recapitulates much of the HCMV pathogenesis, including HCMV-induced cardiovascular diseases. This review provides insight into HCMV-associated cardiovascular diseases and the murine model of MCMV infection, which has been used to study the viral pathogenesis and mechanisms contributing to cardiovascular diseases. Our new functional studies using echocardiography demonstrate tachycardia and hypertrophy in the mouse, similar to HCMV-induced myocarditis in humans. For the first time, we show long term heart dysfunction and that MCMV reactivates from latency in the heart, which raises the intriguing idea that HCMV latency and frequent virus reactivation perturbs long term cardiovascular function.

## 1. Introduction

Human cytomegalovirus (HCMV) is a ubiquitous pathogen that infects 50–90% of the world’s population and causes lifelong infection which, similar to other herpesviruses, can reactivate to produce infectious virus. The prevalence of HCMV is associated with socioeconomic status, nationality, and population density [1,2,3,4]. Unlike infection during immunodeficiency, HCMV infection of immunocompetent individuals is generally asymptomatic but can result in mild febrile illness during primary infection. Despite the lack of symptoms during acute infection, studies point to an association between HCMV seropositivity and an exacerbation of chronic inflammatory diseases later in life [5]. HCMV seropositivity was shown to significantly increase in all-cause mortality in individuals age 25–45 and reduce life expectancy by 4 years in elderly participants when compared to HCMV-seronegative control subjects [6,7]. Long-term HCMV infection stimulates a pro-inflammatory environment, which in turn supports the induction and exacerbation of chronic inflammatory diseases such as autoimmune diseases, some cancers, and cardiovascular diseases [5].

Of particular importance, HCMV seropositivity increases the risk of cardiovascular-induced mortality by 8% in the United States [8,9]. This review will focus on the cardiovascular diseases associated with HCMV infection and will discuss the murine model used to study HCMV-induced cardiovascular pathogenesis. Our lab has recently conducted heart functional studies that demonstrate tachycardia and hypertrophy in MCMV-infected animals during acute and latent infection, further implicating the mouse model of CMV infection with potential to shed new insights on HCMV-induced cardiovascular diseases.

## 2. HCMV and Cardiovascular Diseases

In the early 1970s, HCMV was shown to infect the heart [10]. Since that time, HCMV DNA and proteins have been identified within the heart and vasculature [11]. In vitro, HCMV infects the majority of cell types that reside within the heart and vasculature [12,13]. Seropositivity has been associated with many diseases of the cardiovascular system, including myocarditis, transplant vasculopathy, hypertension, restenosis, and atherosclerosis [11,14,15].

Myocarditis is a heart condition characterized by inflammation of the cardiac muscle that can be caused by infectious agents. While HCMV-induced myocarditis is rare, it can cause life-threatening disease if untreated [9,11]. Sinus tachycardia, ventricular overload, and cardiac hypertrophy can occur in response to tissue damage caused by HCMV infection of the heart [16,17]. Daily treatment with valganciclovir diminishes infection and inflammation within the heart and has been shown to return normal cardiac function within one month of treatment [18].

HCMV infection is a leading cause of morbidity and mortality in transplant recipients for both solid organ and stem cell transplantation [19,20]. This is particularly problematic within the heart where HCMV infection is associated with accelerated cardiac vasculopathy, which significantly reduces allograft survival [21,22,23,24]. This is due to bidirectional damage, in part caused by lytic replication and/or reactivations which damage the tissue, and partly by the indirect cellular response, which stimulates the endothelial and smooth muscle cell proliferation within the heart [25]. Prophylactic HCMV antiviral drug treatment improves survival and reduces transplant rejection; however, drug resistance and toxicity are limitations that can occur during long-term treatment [19,26,27,28]. In addition to the heart, the rejection of many other transplant organs can occur due to HCMV-induced vascular dysfunction [29,30]. In a study of renal transplant recipients, HCMV was associated with higher endothelial stress, carotid intima-media thickness, and integrin expression which resulted in increased cases of atherosclerosis and arteriosclerosis within the kidney [29].

HCMV infection may also impact blood pressure levels. Multiple studies demonstrate a link between HCMV and hypertension, and this correlation seems to be most significant in women and elderly patients [31,32]. In elderly patients over 70, HCMV seropositivity increased systolic blood pressure by 3 mmHg over that seen in HCMV seronegative control patients [33]. Environmental factors and diet may also be factors to consider that increase hypertension during HCMV infection, but further studies are needed to validate this association and determine the viral mechanisms involved.

HCMV also increases the risk of vascular sclerosis including atherosclerosis, arteriosclerosis, and restenosis of the vessels [34,35,36,37,38]. Elevated HCMV-specific IgG antibodies and a loss of naïve T cell populations are known risk factors for HCMV-associated vascular sclerosis [36,38]. HCMV alters wound healing, immune cell infiltration, and induces overactive cell proliferation in the walls of the vasculature, which may contribute to the narrowing of vessels and reduction in blood flow [39,40,41,42,43]. Restenosis is the narrowing of an artery or valve after a corrective surgery has been performed to return blood flow to the vessel. HCMV DNA has been found in approximately one third of restenosis lesions [36,44]. This narrowing is generally stimulated by wound healing and smooth muscle cell proliferation [37]. In addition, immediate-early protein IE84 suppresses p53 function in smooth muscle cells, causing excessive cell proliferation in the areas of damage [34,36].

Overall, there is extensive evidence to support the association of HCMV seropositivity and increased risk of cardiovascular diseases. However, since HCMV is a host restricted pathogen that can only infect humans and human-derived cell lines, mechanistic studies focused on the viral pathogenesis involved are limited. Fortunately, four animal models recapitulate various aspects of the mechanisms of HCMV infection which have contributed a great deal of knowledge on HCMV pathogenesis [45]. Of those four, both rat CMV (RCMV) and murine CMV (MCMV) serve as good small animal models and have been utilized to study HCMV-associated cardiovascular diseases. RCMV shares a great deal of genetic similarity to HCMV and shares many aspects of HCMV-associated atherosclerosis and transplant vasculopathy [30,45,46,47]. However, for the purpose of this review, we will focus on MCMV infection as a model for HCMV-associated cardiovascular diseases.

## 3. MCMV and Cardiovascular Research

Murine models mimic cardiovascular diseases well and have provided a great deal of insight into the pathogenesis of HCMV in the heart and vasculature [48,49,50]. Of the animal models for HCMV, MCMV is the most well-studied and has been used to interrogate viral pathogenesis, immune responses, viral immune evasion, latency, drug discovery, and disease mechanisms, including cardiovascular diseases. MCMV shares ~78% amino acid similarity with HCMV and recapitulates many of the functional aspects of HCMV [51]. Similar to HCMV, MCMV naturally produces acute and latent or persistent infection, as well as causes severe infection in immunocompromised mice [52,53]. Since the heart and vasculature are naturally permissive to MCMV infection, mice are useful tools to study myocarditis, cardiac transplant rejection, hypertension, and vascular sclerosis in the context of infection [48,49,54,55].

Unlike the other HCMV-associated cardiovascular diseases which develop over time, myocarditis onset is sudden and can be associated with primary or persistent infection [48]. Intraperitoneal MCMV inoculation induces acute and chronic myocarditis in immunocompetent mice [48]. In addition, the use of immunocompromised mice has expanded our understanding of chronic myocarditis in immunocompromised populations such as HIV patients [56]. Furthermore, murine IgG antibodies cross react with viral proteins and cardiomyocytes from both wild caught mice and inbred mice, demonstrating that MCMV infection of the heart occurs naturally in the environment, as well as in the laboratory setting [56].

MCMV replication within the heart appears early following intraperitoneal inoculation, with the virus detectable at 3 days post infection (dpi), and peak replication levels occurring at 6–7 dpi. By 10 to 14 dpi, MCMV replication is undetectable in BALB/c mice [48,57]. While viral DNA persistence was shown up to 100 dpi, viral glycoprotein B gene expression was only detectable until 35 dpi, suggesting that the virus enters latency within the heart [58]. Though the exact cell types infected in vivo are unknown, MCMV infection is detected sporadically throughout the heart tissue during replication, as shown by studies using GFP-expressing MCMV viruses [50].

Both HCMV- and MCMV-associated myocarditis results in immune cell infiltration, hypertrophy, and fibrosis [17,18,57,59]. However, it remains unclear whether this damage is predominantly due to viral mechanisms or cellular responses to infection. Both proinflammatory cytokine expression and T cell-mediated killing occurs in vivo, which suggests damage may be associated with the immune response [50]. However, treatment with the antiviral drugs ganciclovir and cidofovir reduces myocarditis in mice similar to humans, suggesting that reduced viral load limits inflammation within the tissue [58]. In addition, MCMV infection within the heart causes a significant increase in IL-2, IL-4, IL-6, IL-10, IL-18, IFN-γ, and TNF-α gene expression early during infection of BALB/c and C57Bl/6 mice [50,60]. However, prolonged IL-18, IFN-γ, and IL-10 expression occurs up to 21 dpi, suggesting that the proinflammatory environment is maintained well after viral replication has ended, and this continued expression is cellular and immune-mediated [50,60]. Furthermore, pre-immunization with viral glycoprotein B inhibits myocarditis development in BALB/c mice indicating that the development of immune memory to MCMV may protect the heart from MCMV-induced myocarditis [61]. Overall, CMV-induced myocarditis results in a proinflammatory environment and damage within the heart that is associated with viral mechanisms and immunological responses to infection.

In transplant patients, HCMV infection is associated with accelerated cardiac vasculopathy, which significantly reduces allograft survival over a five-year period [23]. Similarly, MCMV infection resulted in approximately 80% cardiac allograft rejection by 100 days post transplantation in BALB/c mice [54]. In the transplant recipient mice, MCMV infection caused significantly more fibrosis, immune infiltration, myocyte necrosis, and intimal damage in the heart [54,62]. Although the authors were unable to detect virus reactivation and virus replication by a plaque assay, the use of a focused expansion assay demonstrated increased MCMV DNA levels, suggesting that limited viral reactivation occurred within the grafts. Furthermore, at 21 and 45 dpi, proinflammatory cytokine mRNA expression and a loss of regulatory T cell function were observed in MCMV-infected hearts [54]. In addition to fibrosis-induced damage, vascular sclerosis is a hallmark of cardiac graft rejection [62]. As seen in the development of atherosclerosis, MCMV causes increased intimal cell proliferation, immune cell infiltration, cytokine, and adhesion molecule gene expression within transplanted hearts when compared to uninfected control grafts [62].

Similar to hypertension in elderly HCMV seropositive patients, MCMV infection is sufficient to induce increased blood pressure [55]. This study found that MCMV infection increased levels of IL-6, TNF-a, and MCP-1 proteins in the serum of MCMV-infected mice, and angiotensin 2 protein expression was upregulated in both the serum and venous endothelium [63,64]. Angiotensin 2 regulates renal function as well as blood pressure, and MCMV induction of this hormone may be the mechanism by which hypertension is caused. These studies suggest that CMV-associated inflammation is involved in the increase of blood pressure via the renin-angiotensin pathway, and that both HCMV and MCMV induces high blood pressure by similar mechanisms [55,63,64].

Atherosclerosis, arteriosclerosis, and restenosis are all diseases of the vessels with similar etiology and pathology. Pockets of increased inflammation, immune cell infiltration, and smooth muscle cell proliferation occur within the cellular linings of the vascular walls, which can reduce blood flow and, if ruptured, may cause thrombosis in a downstream vessel. The role of MCMV infection and its link to vascular sclerosis have been studied extensively [65]. In wildtype mice, MCMV, along with a hypercholesterolemic diet, induces arteriole dysfunction and exacerbates lymphocyte and platelet recruitment in the venules. MCMV infection has also been shown to increase P-selectin expression in the heart and lungs of infected mice, which affects lymphocyte recruitment to venules and arteriolar function [65]. Additional studies using IFN-γ knockout mice demonstrated more severe pathology in the vasculature and an inability to maintain latency, suggesting that IFN-γ regulates latency within the vessels and is a major component to controlling MCMV reactivation within the vasculature [66].

Importantly, MCMV infection alters the development of atherosclerosis. Apo E-/- mice have greatly furthered our understanding of the link between MCMV and atherosclerosis and have provided an invaluable tool to study atherosclerosis in the absence of a high cholesterol diet [49,55,67]. Wildtype C57Bl/6J mice develop atherosclerosis on a high cholesterol diet over time, whereas uninfected Apo E-/- mice develop mild atherosclerotic lesions spontaneously by 10 weeks of age without dietary intervention [49]. MCMV-infected wildtype C57Bl/6J mice on a high cholesterol diet had increased severity and kinetics of lesion development. This phenotype is also seen in the Apo E-/- mice infected with MCMV, but with significantly larger and increased number of lesions; however, the lesions were not dependent on a high cholesterol diet. In Apo E-/- mice, MCMV infection induces T cell influx into the aorta. In addition, MCMV infection within the arteries causes macrophage differentiation to a proinflammatory phenotype [67]. These macrophages have increased IL-18 and IFNγ secretions and increased expression of antigen presenting molecules, such as MHC class II, CD40, CD80, and CD86, which further stimulates T cell infiltration [67].

MCMV infection in the arteries also induces MCP-1 gene expression, which increases the infiltration of activated arterial monocytes and T cells. Other atherogenic genes, such as IP-10 and MIG, are upregulated during acute infection in both Apo E-/- mice and wildtype C57Bl/6J mice [68,69]. This suggests that MCMV contributes to the initiation of atherosclerosis through upregulation of inflammatory genes and induces cellular infiltration into the vessels. One pathway that is upregulated by MCMV and appears to play a role in atherosclerosis is the p38 MAP kinase pathway. In MCMV-infected Apo E-/- mice, p38 MAP kinase is upregulated compared to that observed in uninfected Apo E-/- mice and MCMV-infected wildtype C57Bl/6J mice [70]. The p38 protein induces the expression of adhesion molecules, including ICAM-1 and VCAM-1, which increases cell infiltration into the aorta. The inhibition of p38 downregulates adhesion molecules, MCP-1, and proinflammatory cytokines, suggesting this pathway as an important regulator of MCMV-associated atherosclerosis development in these mice.

Apo E-/- mice with and without MCMV infection have also been used in pharmaceutical drugs studies to identify medications that reduce atherosclerosis. For example, MCMV-infected Apo E-/- mice treated with COX2 inhibitors would be predicted to reduce atherosclerotic progression due to a reduction of inflammation. Instead, COX2 treatment increased MCMV susceptibility and worsened atherosclerosis [71]. This study found that inhibiting COX2 caused a proliferative effect in the vasculature and increased lipid accumulation in the aorta, thus suggesting that COX2 inhibitors in HCMV-seropositive patients could result in similar consequences. Due to this result, it may be necessary to test individual drugs for unexpected effects that could occur during MCMV infection.

Together, these studies demonstrate that MCMV infection of the heart and vessels results in damage within the tissue that may promote cardiovascular diseases, including myocarditis, transplant vasculopathy, hypertension, and vascular sclerosis. Through these studies, many cellular, immune, and viral factors that contribute to the progression of these diseases have been identified, making MCMV a useful model for HCMV-associated cardiovascular diseases.

## 4. MCMV and Cardiac Dysfunction

HCMV-associated cardiac dysfunction has clearly been shown in patients with myocarditis, which can present with sinus tachycardia, ventricular overload, and cardiomegaly [18]. All of the studies described above led us to hypothesize that MCMV infection could also cause functional abnormalities in the heart. Thus, we inoculated mice with MCMV and performed echocardiography at acute and latent time points after infection [57]. Echocardiography is a technique performed in clinical practice to assess heart function by using sound waves to visualize the heart beating in real time. This allows for quantitative measurements pertaining to heart rate and left ventricular function based on parameters, including internal diameter, posterior wall thickness, end volume, stroke volume, ejection fraction, and fraction shortening during diastolic and systolic phases of beating [72].

To measure MCMV-induced dysfunction, BALB/c mice were inoculated i.p. with 1 × 10^6^ PFU and evaluated by echocardiography at 14 dpi, an acute time point, and 50 dpi, a time when MCMV has established latency [57,73]. At both time points, MCMV-infected mice had significantly higher heart rates (437 and 447 beats per minute, respectively) than uninfected control animals (350–375 beats per minute) [57]. Strikingly, an aberrant rhythm of heart beating was also observed in the MCMV-infected animals. At 50 dpi, MCMV infection also resulted in significantly increased left ventricular posterior wall thickness. This parameter, along with fibrosis seen in the hearts by Masson’s Trichrome staining, suggested hypertrophy of the left ventricle. Since none of the other parameters analyzed by echocardiography were altered, MCMV-infected animals were still able to maintain cardiac output. However, tachycardia, hypertrophy, and fibrosis are known compensatory mechanisms of the heart in response to tissue damage [74,75]. In order to preserve cardiac structure and function, interstitial fibrosis replaces dead cells in damaged areas to ensure necessary blood flow throughout the body. This, however, results in increased heart rate and muscle thickness. Over time, these changes, which are used to maintain pressure, stroke volume, and ejection fraction, result in replacement fibrosis, which is more rigid and reduces the heart’s capacity to expand and contract [74,75]. This suggests that tissue damage observed early following MCMV infection also persists long after the primary disease.

In addition to dysfunction, macroscopic alterations on the outer epicardium of the MCMV-infected hearts was observed at both 14 and 50 dpi [50,57,76]. These areas were previously determined to be calcification although no assays were performed to verify this [50]. In our study, Masson’s trichrome staining revealed collagen accumulation in these areas [57]. Additional histology analysis on the hearts using Alizarin red solution, which stains areas containing calcium, revealed that calcium and collagen appear to co-localize at these locations (data in preparation) [77]. In uninfected BALB/c mice, small amounts of calcification occurs spontaneously throughout the course of their lifespan, however, MCMV infection significantly exacerbates this phenotype [57,76].

Since MCMV establishes long term latent infection in a number of tissues, cardiac damage in the heart could occur when virus reactivates from latency [48,54]. Viral DNA maintenance and increased viral DNA levels have been detected by PCR during latency; however, progeny virus production due to MCMV reactivation has not been demonstrated [48,54]. Therefore, we asked whether MCMV can undergo reactivation in the heart. We hypothesized that the cardiac dysfunction during infection was exacerbated in latently infected mice by repeated reactivations in the heart throughout the life of the animal. Following echocardiography at 50 dpi, MCMV-infected hearts were evaluated by an explant reactivation assay. This assay consists of culturing primary tissues ex vivo for 6 weeks to evaluate MCMV reactivation in the explanted tissue culture [78]. Herpesvirus latency is defined as detection of viral DNA with no virus progeny production and the ability to reactivate from latency [79]. Using this assay, we demonstrated that, despite the low viral replication shown in the heart during acute infection, MCMV readily reactivated from the heart at 50 dpi, similar to other latently infected tissues such as the spleen or lung [58]. This data, along with the exacerbated fibrosis and dysfunction that is found at 50 dpi, suggests that repeated reactivations could contribute to long term damage and dysfunction in the heart.

In our studies, mice did not die from MCMV infection; thus, we questioned whether this dysfunction was merely transient or continued beyond 50 dpi. For these studies, mice were evaluated by echocardiography at 90 dpi. At this time point, there were no longer significant differences in calcification or heart rate when compared to control animals, suggesting that some features of damage and dysfunction resolve over time (data in preparation) [77]. Conversely, the significant increase in fibrosis and hypertrophy was still observed at 90 dpi. In addition, left ventricular internal diameter was significantly reduced in MCMV-infected animals at this time point. This suggests that the hypertrophy had progressed and muscle had expanded into the ventricle, thus decreasing the diameter (data in preparation) [77]. While cardiac output was still being maintained, a reduction in internal diameter suggests that less blood was moving in and out of the ventricle during each heartbeat. Reactivation at 90 dpi also showed similar percent reactivation and viral progeny production as detected at 50 dpi (data in preparation) [77]. Together, these results suggest that cardiac dysfunction is not a transient effect during MCMV infection and that damage and dysfunction continues long term within the heart.

## 5. Conclusions

**Figure 1:**
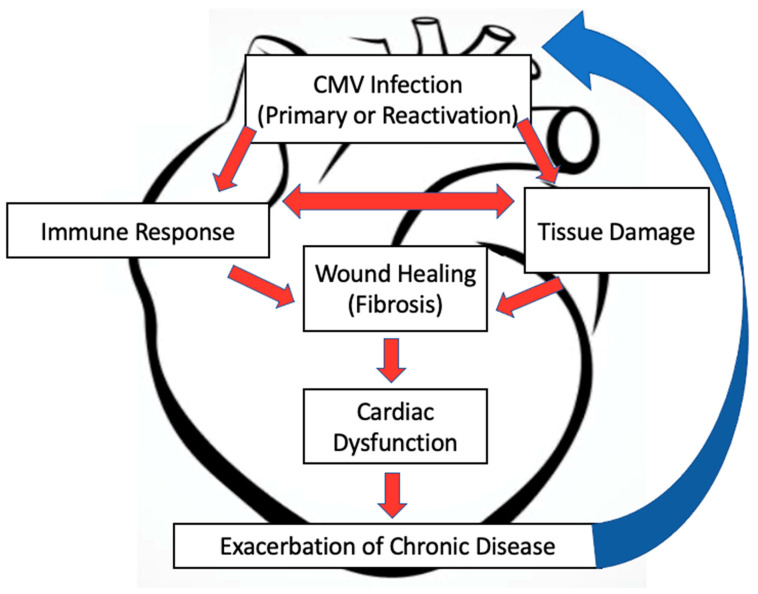
Summary diagram of CMV-associated damage and dysfunction within the heart.

HCMV causes lifelong latent infection that has been associated with many morbidities in humans, including diseases of the cardiovascular system. Long term HCMV seropositivity is associated with an 8% increased risk in cardiovascular-induced mortality in the United States [9]. Primary HCMV infection in immunocompetent individuals or in immunocompromised patients can result in life threatening functional abnormalities within the heart, including sinus tachycardia, ventricular overload, and hypertrophy [16,17,18]. Given the current knowledge, we postulate that CMV reactivates within the heart and vasculature, which induces repeated changes within the microenvironment of the tissue that, over time, exacerbate cardiovascular diseases. Using the MCMV model, we show similar dysfunction and now point perhaps to a role for latent infection and reactivation events in the involvement of long term heart dysfunction [57]. As shown in Figure 1, MCMV infection and reactivation could stimulate chronic changes and tissue damage in the heart, contributed by altered inflammatory cytokine expression, modulation of signaling pathways, induction of immune cell infiltration, or activation of virus-specific tissue resident memory T cells. Additionally, the proinflammatory environment could trigger CMV reactivation within the tissue, creating a vicious cycle of repeated virus activity and cellular responses which promotes and exacerbates disease. Based on the sequence homology between HCMV and MCMV and the similar dysfunction observed within the heart, MCMV is an ideal model for future studies evaluating HCMV-associated cardiovascular diseases and dysfunction.

Further studies are needed to determine which in vivo cell types become infected in the heart, especially to identify those cell types which harbor latent genomes. The mechanisms involved in cardiac damage and dysfunction are largely unknown. It is possible that both viral and host factors contribute to the associated damage within the heart and, to fully understand this pathology, extensive experimental analysis of both is required. However, since HCMV infection induces both cellular and extracellular remodeling, it is likely that viral genes contribute to altering the microenvironment of the heart [41,42,80]. This could include virally encoded G-protein coupled receptor (vGPCR) homologs, as they have been shown to modulate cytokine expression, alter cell signaling, and influence cellular migration. Both the HCMV-encoded vGPCR, US28, and the MCMV-encoded functional homolog, M33, activate multiple signaling pathways, which could modulate downstream cellular functions and contribute to the changes that occur in the heart [81]. A recent publication demonstrated in aortic allograft transplants that the absence of M33 during MCMV infection results in altered cell proliferation, adhesion molecule expression, and immune cell infiltration, thus suggesting that the vGPCRs could influence cardiac damage and dysfunction [82]. Current studies in our lab are underway to determine various viral and host factors which contribute to cardiac dysfunction following MCMV infection. Our preliminary results indicate that modulation of the heart microenvironment, e.g., cytokines, extracellular matrix, and growth factors, occurs differentially following wildtype MCMV vs. M33-deficient virus infection. Therefore, we propose studies focusing on the cell types infected in vivo, the immune responses occurring in the heart, and the viral mechanisms at play as future directions that could improve our understanding of damage and dysfunction in the heart and contribute to the development of novel therapeutic strategies to treat heart disease in HCMV-seropositive individuals.

## References

[1] Staras S.A.S., Flanders W.D., Dollard S.C., Pass R.F., McGowan J.E., Cannon M.J. (2008). Cytomegalovirus Seroprevalence and Childhood Sources of Infection: A Population-Based Study among Pre-Adolescents in the United States. J. Clin. Virol..

[2] Zuhair M., Smit G.S.A., Wallis G., Jabbar F., Smith C., Devleesschauwer B., Griffiths P. (2019). Estimation of the Worldwide Seroprevalence of Cytomegalovirus: A Systematic Review and Meta-Analysis. Rev. Med. Virol..

[3] Bate S.L., Dollard S.C., Cannon M.J. (2010). Cytomegalovirus Seroprevalence in the United States: The National Health and Nutrition Examination Surveys, 1988–2004. Clin. Infect. Dis..

[4] Cannon M.J., Schmid D.S., Hyde T.B. (2010). Review of Cytomegalovirus Seroprevalence and Demographic Characteristics Associated with Infection. Rev. Med. Virol..

[5] Soderberg-Naucler C. (2006). Does Cytomegalovirus Play a Causative Role in the Development of Various Inflammatory Diseases and Cancer?. J. Intern. Med..

[6] Vescovini R., Telera A.R., Pedrazzoni M., Abbate B., Rossetti P., Verzicco I., Arcangeletti M.C., Medici M.C., Calderaro A., Volpi R. (2016). Impact of Persistent Cytomegalovirus Infection on Dynamic Changes in Human Immune System Profile. PLoS ONE.

[7] Savva G.M., Pachnio A., Kaul B., Morgan K., Huppert F.A., Brayne C., Moss P.A.H. (2013). The Medical Research Council Cognitive Function and Ageing Study Cytomegalovirus Infection Is Associated with Increased Mortality in the Older Population. Aging Cell.

[8] Okedele O.O., Nelson H.H., Oyenuga M.L., Thyagarajan B., Prizment A. (2020). Cytomegalovirus and Cancer-Related Mortality in the National Health and Nutritional Examination Survey. Cancer Causes Control.

[9] Simanek A.M., Dowd J.B., Pawelec G., Melzer D., Dutta A., Aiello A.E. (2011). Seropositivity to Cytomegalovirus, Inflammation, All-Cause and Cardiovascular Disease-Related Mortality in the United States. PLoS ONE.

[10] Wilson R.S.E., Morris T.H., Rees J.R. (1972). Cytomegalovirus Myocarditis. Br. Heart J..

[11] Petrie B.L., Melnick J.L., Adam E., Burek J., McCollum C.H., DeBakey M.E. (1987). Nucleic Acid Sequences of Cytomegalovirus in Cells Cultured from Human Arterial Tissue. J. Infect. Dis..

[12] Sinzger C., Grefte A., Plachter B., Gouw A.S.H., The T.H., Jahn G. (1995). Fibroblasts, Epithelial Cells, Endothelial Cells and Smooth Muscle Cells Are Major Targets of Human Cytomegalovirus Infection in Lung and Gastrointestinal Tissues. J. Gen. Virol..

[13] Soderberg-Naucler C., Larsson S., Bergstedt-Lindqvist S., Moller E. (1993). Identification of Blood Mononuclear Cells Permissive of Cytomegalovirus Infection in Vitro. Transplant. Proc..

[14] Schönian U., Crombach M., Maisch B. (1993). Assessment of Cytomegalovirus DNA and Protein Expression in Patients with Myocarditis. Clin. Immunol. Immunopathol..

[15] Melnick J. (1983). Cytomegalovirus Antigen within Human Arterial Smooth Muscle Cells. Lancet.

[16] Kyto V., Vuorinen T., Saukko P., Lautenschlager I., Lignitz E., Saraste A., Voipio-Pulkki L.-M. (2005). Cytomegalovirus Infection of the Heart Is Common in Patients with Fatal Myocarditis. Clin. Infect. Dis..

[17] Magno Palmeira M., Umemura Ribeiro H.Y., Garcia Lira Y., Machado Jucá Neto F.O., da Silva Rodrigues I.A., Fernandes da Paz L.N., da Nascimento Pinheiro M.C. (2016). Heart Failure Due to Cytomegalovirus Myocarditis in Immunocompetent Young Adults: A Case Report. BMC Res. Notes.

[18] Padala S.K., Kumar A., Padala S. (2014). Fulminant Cytomegalovirus Myocarditis in an Immunocompetent Host: Resolution with Oral Valganciclovir. Texas Heart Inst. J..

[19] Kotton C.N., Kumar D., Caliendo A.M., Huprikar S., Chou S., Danziger-Isakov L., Humar A. (2018). The Third International Consensus Guidelines on the Management of Cytomegalovirus in Solid-Organ Transplantation. Transplantation.

[20] Campos C.F., Leite L., Pereira P., Vaz C.P., Branca R., Campilho F., Freitas F., Ligeiro D., Marques A., Torrado E. (2019). PTX3 Polymorphisms Influence Cytomegalovirus Reactivation After Stem-Cell Transplantation. Front. Immunol..

[21] Grattan M., Moreno-Cabral C., Starnes V., Oyer P., Stinson E.B., Shumway N. (1989). Cytomegalovirus Infection Is Associated with Cardiac Allograft Rejection and Atherosclerosis. JAMA.

[22] Valantine H.A., Gao S.-Z., Menon S.G., Renlund D.G., Hunt S.A., Oyer P., Stinson E.B., Brown B.W., Merigan T.C., Schroeder J.S. (1999). Impact of Prophylactic Immediate Posttransplant Ganciclovir on Development of Transplant Atherosclerosis: A Post Hoc Analysis of a Randomized, Placebo-Controlled Study. Circulation.

[23] Weill D. (2001). Role of Cytomegalovirus in Cardiac Allograft Vasculopathy. Transpl. Infect. Dis..

[24] Johansson I., Andersson R., Friman V., Selimovic N., Hanzen L., Nasic S., Nyström U., Sigurdardottir V. (2015). Cytomegalovirus Infection and Disease Reduce 10-Year Cardiac Allograft Vasculopathy-Free Survival in Heart Transplant Recipients. BMC Infect. Dis..

[25] Koskinen P.K., Kallio E.A., Tikkanen J.M., Sihvola R.K., Hayry P.J., Lemstrom K.B. (1999). Cytomegalovirus Infection and Cardiac Allograft Vasculopathy. Transpl. Infect. Dis..

[26] Merigan T.C., Renlund D.G., Keay S., Bristow M., Starnes V., O’Connell J., Resta S., Dunn D., Gamberg P., Ratkovec R. (1992). A Controlled Trial of Ganciclovir to Prevent Cytomegalovirus Disease after Heart Transplantation. N. Engl. J. Med..

[27] Streblow D., Orloff S., Nelson J. (2007). Acceleration of Allograft Failure by Cytomegalovirus. Curr. Opin. Immunol..

[28] Atabani S.F., Smith C., Atkinson C., Aldridge R.W., Rodriguez-Perálvarez M., Rolando N., Harber M., Jones G., O’Riordan A., Burroughs A.K. (2012). Cytomegalovirus Replication Kinetics in Solid Organ Transplant Recipients Managed by Preemptive Therapy: CMV and Preemptive Antiviral Therapy. Am. J. Transplant..

[29] Lee S., Brook E., Affandi J., Howson P., Tanudjaja S.A., Dhaliwal S., Irish A., Price P. (2019). A High Burden of Cytomegalovirus Marks Poor Vascular Health in Transplant Recipients More Clearly than in the General Population. Clin. Transl. Immunol..

[30] Streblow D.N., Kreklywich C.N., Andoh T., Moses A.V., Dumortier J., Smith P.P., Defilippis V., Fruh K., Nelson J.A., Orloff S.L. (2008). The Role of Angiogenic and Wound Repair Factors During CMV-Accelerated Transplant Vascular Sclerosis in Rat Cardiac Transplants: CMV Accelerated TVS Involves Angiogenesis and Wound Healing. Am. J. Transplant..

[31] Li S., Zhu J., Zhang W., Chen Y., Zhang K., Popescu L.M., Ma X., Bond Lau W., Rong R., Yu X. (2011). Signature MicroRNA Expression Profile of Essential Hypertension and Its Novel Link to Human Cytomegalovirus Infection. Circulation.

[32] Li C., Samaranayake N.R., Ong K.L., Wong H.K., Cheung B.M.Y. (2012). Is Human Cytomegalovirus Infection Associated with Hypertension? The United States National Health and Nutrition Examination Survey 1999–2002. PLoS ONE.

[33] Firth C., Harrison R., Ritchie S., Wardlaw J., Ferro C.J., Starr J.M., Deary I.J., Moss P. (2016). Cytomegalovirus Infection Is Associated with an Increase in Systolic Blood Pressure in Older Individuals. QJM.

[34] Speir E., Modali R., Huang E.-S., Leon M.B., Shawl F., Finkel T., Epstein S.E. (1994). Potential Role of Human Cytomegalovirus and P53 Interaction in Coronary Restenosis. Science.

[35] Dummer S., Lee A., Breinig M., Kormos R., Ho M., Griffiths B. (1994). Investigation of Cytomegalovirus-Infection as a Risk Factor for Coronary Atherosclerosis in the Explanted Hearts of Patients Undergoing Heart-Transplantation. J. Med. Virol..

[36] Zhou Y.F., Finkel T. (1996). Association between Prior Cytomegalovirus Infection and the Risk of Restenosis after Coronary Atherectomy. N. Engl. J. Med..

[37] Blum A., Peleg A., Weinberg M. (2003). Anti-Cytomegalovirus (CMV) IgG Antibody Titer in Patients with Risk Factors to Atherosclerosis. Clin. Exp. Med..

[38] Olson N.C., Doyle M.F., Jenny N.S., Huber S.A., Psaty B.M., Kronmal R.A., Tracy R.P. (2013). Decreased Naive and Increased Memory CD4+ T Cells Are Associated with Subclinical Atherosclerosis: The Multi-Ethnic Study of Atherosclerosis. PLoS ONE.

[39] Streblow D.N., Soderberg-Naucler C., Vieira J., Smith P., Wakabayashi E., Ruchti F., Mattison K., Altschuler Y., Nelson J.A. (1999). The Human Cytomegalovirus Chemokine Receptor US28 Mediates Vascular Smooth Muscle Cell Migration. Cell.

[40] Reinhardt B., Mertens T., Mayrbeyrle U., Frank H., Luske A., Schierling K., Waltenberger J. (2005). HCMV Infection of Human Vascular Smooth Muscle Cells Leads to Enhanced Expression of Functionally Intact PDGF β-Receptor. Cardiovasc. Res..

[41] Reinhardt B. (2006). Human Cytomegalovirus-Induced Reduction of Extracellular Matrix Proteins in Vascular Smooth Muscle Cell Cultures: A Pathomechanism in Vasculopathies?. J. Gen. Virol..

[42] Dumortier J., Streblow D.N., Moses A.V., Jacobs J.M., Kreklywich C.N., Camp D., Smith R.D., Orloff S.L., Nelson J.A. (2008). Human Cytomegalovirus Secretome Contains Factors That Induce Angiogenesis and Wound Healing. JVI.

[43] Beyaz M.O., Ugurlucan M., Oztas D.M., Meric M., Conkbayir C., Agacfidan A., Onel M., Alpagut U. (2019). Evaluation of the Relationship between Plaque Formation Leading to Symptomatic Carotid Artery Stenosis and Cytomegalovirus by Investigating the Virus DNA. AMSAD.

[44] Chen R., Xiong S., Yang Y., Fu W., Wang Y., Ge J., Gilchrist J.S.C., Tappia P.S., Netticadan T. (2003). The relationship between human cytomegalovirus infection and atherosclerosis development. Biochemistry of Diabetes and Atherosclerosis.

[45] Dogra P., Sparer T.E., Yurochko A.D., Miller W.E. (2014). What We Have Learned from Animal Models of HCMV. Human Cytomegaloviruses.

[46] Vink C., Beuken E., Bruggeman C.A. (2000). Complete DNA Sequence of the Rat Cytomegalovirus Genome. J. Virol..

[47] Price P., Olver S.D. (1996). Syndromes Induced by Cytomegalovirus Infection. Clin. Immunol. Immunopathol..

[48] Lenzo J.C., Fairweather D., Cull V., Shellam G.R., James(Lawson) C.M. (2002). Characterisation of Murine Cytomegalovirus Myocarditis: Cellular Infiltration of the Heart and Virus Persistence. J. Mol. Cell. Cardiol..

[49] Vliegen I., Duijvestijn A., Grauls G., Herngreen S., Bruggeman C., Stassen F. (2004). Cytomegalovirus Infection Aggravates Atherogenesis in ApoE Knockout Mice by Both Local and Systemic Immune Activation. Microbes Infect..

[50] Ritter J.T., Tang-Feldman Y.J., Lochhead G.R., Estrada M., Lochhead S., Yu C., Ashton-Sager A., Tuteja D., Leutenegger C., Pomeroy C. (2010). In Vivo Characterization of Cytokine Profiles and Viral Load during Murine Cytomegalovirus-Induced Acute Myocarditis. Cardiovasc. Pathol..

[51] Rawlinson W.D., Farrell H.E., Barrell B.G. (1996). Analysis of the Complete DNA Sequence of Murine Cytomegalovirus. J. Virol..

[52] Reddehase M.J., Podlech J., Grzimek N.K.A. (2002). Mouse Models of Cytomegalovirus Latency: Overview. J. Clin. Virol..

[53] Weisblum Y., Panet A., Haimov-Kochman R., Wolf D.G. (2014). Models of Vertical Cytomegalovirus (CMV) Transmission and Pathogenesis. Semin. Immunopathol..

[54] Cook C.H., Bickerstaff A.A., Wang J.-J., Zimmerman P.D., Forster M.R., Nadasdy T., Colvin R.B., Hadley G.A., Orosz C.G. (2008). Disruption of Murine Cardiac Allograft Acceptance by Latent Cytomegalovirus: Disruption of Murine Cardiac Allograft. Am. J. Transplant..

[55] Cheng J., Ke Q., Jin Z., Wang H., Kocher O., Morgan J.P., Zhang J., Crumpacker C.S. (2009). Cytomegalovirus Infection Causes an Increase of Arterial Blood Pressure. PLoS Pathog..

[56] Lawson C.M., O’Donoghue H., Bartholomaeus W.N., Reed W.D. (1990). Genetic Control of Mouse Cytomegalovirus-Induced Myocarditis. Immunology.

[57] Bonavita C.M., White T.M., Francis J., Cardin R.D. (2020). Heart Dysfunction Following Long-Term Murine Cytomegalovirus Infection: Fibrosis, Hypertrophy, and Tachycardia. Viral Immunol..

[58] Lenzo J.C., Shellam G.R., Lawson C.M. (2001). Ganciclovir and Cidofovir Treatment of Cytomegalovirus-Induced Myocarditis in Mice. Antimicrob. Agents Chemother..

[59] Iglezias S.D., Benvenuti L.A., Calabrese F., Salemi V.M.C., Silva A.M.G., Carturan E., de Oliveira S.A., Thiene G., De Brito T. (2008). Endomyocardial Fibrosis: Pathological and Molecular Findings of Surgically Resected Ventricular Endomyocardium. Virchows Arch..

[60] Lenzo J.C., Mansfield J.P., Sivamoorthy S., Cull V.S., James C.M. (2003). Cytokine Expression in Murine Cytomegalovirus-Induced Myocarditis: Modulation with Interferon-α Therapy. Cell. Immunol..

[61] Cull V.S., Broomfield S., Bartlett E.J., Brekalo N.L., James C.M. (2002). Coimmunisation with Type I IFN Genes Enhances Protective Immunity against Cytomegalovirus and Myocarditis in GB DNA-Vaccinated Mice. Gene Ther..

[62] Heim C., Abele-Ohl S., Eckl S., Ramsperger-Gleixner M., Mahmoudian S., Weyand M., Stamminger T., Ensminger S.M. (2010). Murine Cytomegalovirus Infection Leads to Increased Levels of Transplant Arteriosclerosis in a Murine Aortic Allograft Model. Transplantation.

[63] Khoretonenko M.V., Leskov I.L., Jennings S.R., Yurochko A.D., Stokes K.Y. (2010). Cytomegalovirus Infection Leads to Microvascular Dysfunction and Exacerbates Hypercholesterolemia-Induced Responses. Am. J. Pathol..

[64] Senchenkov E., Khoretonenko M.V., Leskov I.L., Ostanin D.V., Stokes K.Y. (2011). P-Selectin Mediates the Microvascular Dysfunction Associated with Persistent Cytomegalovirus Infection in Normocholesterolemic and Hypercholesterolemic Mice: P-Selectin in Persistent CMV Infection. Microcirculation.

[65] Presti R.M., Pollock J.L., Canto A.J.D., O’Guin A.K. (1998). Interferon Regulates Acute and Latent Murine Cytomegalovirus Infection and Chronic Disease of the Great Vessels. J. Exp. Med..

[66] Vliegen I., Stassen F., Grauls G., Blok R., Bruggeman C. (2002). MCMV Infection Increases Early T-Lymphocyte Influx in Atherosclerotic Lesions in ApoE Knockout Mice. J. Clin. Virol..

[67] Vliegen I., Duijvestijn A., Stassen F., Bruggeman C. (2004). Murine Cytomegalovirus Infection Directs Macrophage Differentiation into a Pro-Inflammatory Immune Phenotype: Implications for Atherogenesis. Microbes Infect..

[68] Froberg M.K., Adams A., Seacotte N., Parker-Thornburg J., Kolattukudy P. (2001). Cytomegalovirus Infection Accelerates Inflammation in Vascular Tissue Overexpressing Monocyte Chemoattractant Protein-1. Circ. Res..

[69] Burnett M.S., Durrani S., Stabile E., Saji M., Lee C.W., Kinnaird T.D., Hoffman E.P., Epstein S.E. (2004). Murine Cytomegalovirus Infection Increases Aortic Expression of Proatherosclerotic Genes. Circulation.

[70] Tang-Feldman Y.J., Lochhead S.R., Lochhead G.R., Yu C., George M., Villablanca A.C., Pomeroy C. (2013). Murine Cytomegalovirus (MCMV) Infection Upregulates P38 MAP Kinase in Aortas of Apo E KO Mice: A Molecular Mechanism for MCMV-Induced Acceleration of Atherosclerosis. J. Cardiovasc. Trans. Res..

[71] Rott D., Zhu J., Burnett M.S., Zhou Y., Zalles-Ganley A., Ogunmakinwa J., Epstein S.E. (2003). Effects of MF-Tricyclic, a Selective Cyclooxygenase-2 Inhibitor, on Atherosclerosis Progression and Susceptibility to Cytomegalovirus Replication in Apolipoprotein-E Knockout Mice. J. Am. Coll. Cardiol..

[72] Basman C., Parmar Y.J., Kronzon I. (2017). Intracardiac Echocardiography for Structural Heart and Electrophysiological Interventions. Curr. Cardiol. Rep..

[73] Cardin R.D., Schaefer G.C., Allen J.R., Davis-Poynter N.J., Farrell H.E. (2009). The M33 Chemokine Receptor Homolog of Murine Cytomegalovirus Exhibits a Differential Tissue-Specific Role during In Vivo Replication and Latency. JVI.

[74] Rai V., Sharma P., Agrawal S., Agrawal D.K. (2017). Relevance of Mouse Models of Cardiac Fibrosis and Hypertrophy in Cardiac Research. Mol. Cell Biochem..

[75] Suthahar N., Meijers W.C., Silljé H.H.W., de Boer R.A. (2017). From Inflammation to Fibrosis—Molecular and Cellular Mechanisms of Myocardial Tissue Remodelling and Perspectives on Differential Treatment Opportunities. Curr. Heart Fail. Rep..

[76] Glass A.M., Coombs W., Taffet S.M. (2013). Spontaneous Cardiac Calcinosis in BALB/CByJ Mice. Comp. Med..

[77] Bonavita C.M. (2021).

[78] Almanan M., Raynor J., Sholl A., Wang M., Chougnet C., Cardin R.D., Hildeman D.A. (2017). Tissue-Specific Control of Latent CMV Reactivation by Regulatory T Cells. PLoS Pathog..

[79] Roizman B., Sears A. (1987). An Inquiry Into The Mechanisms Of Herpes Simplex Virus Latency. Annu. Rev. Microbiol..

[80] Hertel L., Mocarski E.S. (2004). Global Analysis of Host Cell Gene Expression Late during Cytomegalovirus Infection Reveals Extensive Dysregulation of Cell Cycle Gene Expression and Induction of Pseudomitosis Independent of US28 Function. JVI.

[81] Farrell H.E., Abraham A.M., Cardin R.D., Sparre-Ulrich A.H., Rosenkilde M.M., Spiess K., Jensen T.H., Kledal T.N., Davis-Poynter N. (2011). Partial Functional Complementation between Human and Mouse Cytomegalovirus Chemokine Receptor Homologues. J. Virol..

[82] Fritz N.M., Stamminger T., Ramsperger-Gleixner M., Kuckhahn A.V., Müller R., Weyand M., Heim C. (2021). Cytomegalovirus Chemokine Receptor M33 Knockout Reduces Chronic Allograft Rejection in a Murine Aortic Transplant Model. Transplant. Immunol..

